# EHAC medical working group best practice advice on the role of air rescue and pre hospital critical care at major incidents

**DOI:** 10.1186/s13049-018-0522-1

**Published:** 2018-08-15

**Authors:** Julian Thompson, Marius Rehn, Stephen J. M. Sollid, on behalf of the European HEMS and Air Ambulance Committee (EHAC)

**Affiliations:** 1 Adult Intensive Care Unit, North Bristol NHS Trust, Bristol, UK; 2 Great Western Air Ambulance, Bristol, UK; 3 Severn Major Trauma Network, North Bristol NHS Trust, Bristol, UK; 4 Department of Research, Norwegian Air Ambulance Foundation, Drøbak, Norway; 5 Pre-hospital Division, Air Ambulance Department, Oslo University Hospital, Oslo, Norway; 6 Department of Health Studies, University of Stavanger, Stavanger, Norway; 7 Air Ambulance Department, Oslo University Hospital, Oslo, Norway

## Abstract

**Background:**

Helicopter EMS (HEMS) teams may perform a variety of clinical, managerial and transport functions during major incident management. Despite national and international variations in HEMS systems, the rapid delivery of HEMS personnel with advanced skills in major incident management and clinical scene leadership has been crucial to the delivery of an effective medical response at previous incidents. This document outlines the Best Practice Advice of the European HEMS and Air Ambulance Committee (EHAC) Medical Working Group on how HEMS and Pre Hospital Critical Care teams may maximise the positive impact of their resources in the event of Major Incidents.

**Methods:**

Narrative literature review and expert consensus.

**Results:**

To ensure a safe, coordinated and effective response, HEMS teams require suitable, proportionate and up to date major incident plans that are integrated into the major incident plans of other regional emergency and healthcare services. Role specific protocols, training and equipment should be adapted to the expected HEMS role in the major incident plan and likely regional threats. System and incident factors will influence HEMS utilisation during the major incident response and can include patient and staff transfer, equipment resupply, aerial assessment, search and rescue, clinical leadership and advanced care. During the recovery phase of a major incident there is a need to ensure restoration of conventional service and address the welfare of involved HEMS personnel. Standardised reporting of major incidents is strongly recommended for clinical governance, legal and research reasons.

**Conclusions:**

The rapid delivery of HEMS personnel with advanced skills in Major Incident management and clinical scene leadership is crucial to the delivery of an effective medical response at Major Incidents.

## Background

A major incident can be defined as an incident that requires the mobilisation of extraordinary emergency medical service (EMS) resources and is identified as a major incident in that system [1, 2]. Major incidents cause a terrible global burden of fatalities, injury, disruption and expense, but for individual EMS they are infrequent and present extreme clinical and logistical challenges.

Helicopter EMS (HEMS) teams may perform a variety of clinical, managerial and transport functions during major incident management. Helicopters have the capability to bring specialised teams and equipment to incident scenes, transport patients, provide search and rescue services, and perform overhead surveillance. When a site is remote or conventional access routes are compromised, helicopters may be the only way to transport personnel, equipment and patients to and from an incident [3].

HEMS services differ nationally and internationally in the medical expertise of team members, aviation platform, funding model and integration into state EMS. Despite these variations, the rapid delivery of HEMS personnel with advanced skills in Major Incident management and clinical scene leadership has been crucial to the delivery of an effective medical response at previous Major Incidents [4–6].

This document outlines the Best Practice Advice of the EHAC Medical Working Group on how HEMS and Pre Hospital Critical Care teams may maximise the positive impact of their resources in the event of Major Incidents. This document is intended to compliment and not supplant existing local and international Major Incident guidance.

Fixed-wing Air Ambulance rarely have a role in the initial response phase of Major Incidents, but are often used for secondary/interhospital transport to relieve local hospital capacity or transport patients to definitive care in the development and conclusion phase of a Major Incident response. The EHAC Medical Working Group have decided to not describe fixed-wing Air Ambulance separately in this document, but recommend that the same Best Practice Advise be valid for fixed-wing as for HEMS.

## Best practice advice on major incident management

### Preparation

#### Major incident plans

- HEMS units require a suitable, proportionate and up to date Major Incident plan that includes medical, aviation, and technical aspects. Further, support services e.g. communication, fuel and resupply should also be integrated into the service plan
- Local Major Incident plans should include clear pre-defined roles, responsibilities and command of HEMS medical personnel and helicopter utilisation
- HEMS Major Incident plans must be integrated with the Major Incident plans of other local medical and non-medical emergency services including fire, police and military
- Information on capability, capacity, landing site and contact details should be available to HEMS teams and their control centres for regional and supra-regional hospitals to facilitate patient distribution and optimise surge capacity
- Depending upon regional threat, HEMS teams may require formal planning cooperation with tactical law enforcement units to ensure team safety, helicopter base security and efficient clinical care in non permissive environments
- Consider maintaining units in reserve or delaying HEMS deployment until incidents are declared to avoid HEMS resources from becoming fully committed to potential or minor incidents at the expense of casualties at decompensated sites. Reserve assets also maintain the ability to respond to multiple incident sites and ensure that conventional activity can be fully resumed at the conclusion of the incident [6]
- Ensure robust communication methods between HEMS teams and other emergency services exist to allow interoperability and a cohesive response
- Establish efficient communication cascades within HEMS systems to ensure teams can be mobilised and coordinated in the event of a major incident

#### Training

- HEMS medical personnel must complete a recognised Major Incident management training course that allows operational integration with local emergency services
- HEMS operational staff must ensure that they are familiar with the HEMS and local Major Incident plans
- Major Incident exercises should be conducted on a regular basis together with other rescue services, in accordance with local statutory requirements, to confirm operational familiarity with plans and identify deficits [7]
- Training in tactical, chemical, biological or radiation operational safety should be provided to HEMS teams that are expected to provide immediate patient triage and care or if no alternative tactical medical team exists [8], [9]

### Equipment

- Anticipate the provision of equipment and drug requirements of mass casualty incidents sufficient to deploy multiple equipped HEMS teams in exceptional circumstances [4]
- Provide Personal Protective Equipment (PPE) to allow safe working in a Major Incident environment with conventional scene hazards
- Risk evaluate for the likelihood of Chemical, Biological, Radiation, Nuclear or Explosive (CBRNE), ballistic or specific local scene hazards (temperature, immersion, etc) and consider stocking sufficient risk specific PPE, logistics and detection equipment to allow the safe deployment of multiple teams
- Consider the routine carriage of back-up communications equipment due to the multifactorial potential for conventional communication network failure
- Prepare items such as triage labels, action cards, event logs, etc. in accordance with local protocols to facilitate the organisation and documentation of Major Incidents

### Support services

- Support structures like maintenance, supplies, transfusion services, human resources, public relations and communications departments are all integrated parts of operational HEMS units and should be embedded in major incident plans to maintain resilience in prolonged operations.

## EHAC MWG statement

Major incident planning, training, resources and exercises are not only central to the delivery of an effective emergency response but are legal requirements in some countries http://www.legislation.gov.uk/ukpga/2004/36/contents. Moreover, healthcare organisations have a legal duty of care for their employees and must appropriately equip and prepare them to work in such rare, high risk but foreseeable circumstances. Failure to adequately train or protect staff has resulted in severe short and long term health consequences for medical staff at incidents such as the sarin attacks in Tokyo [10, 11] and following the collapse of the World Trade Centre, New York on 9/11 2001 [12]. Conversely, specialist training and equipment allowed medical personnel to effectively operate in the ballistic environment during the November 2014 Paris attacks and deliver emergency patient care far earlier than is conventional in most systems [9].

## Response

The medical management of a Major Incident will be guided by local and international protocols [1]. The focus of the BPA below is upon the specific benefit and role of HEMS teams in the wider Major Incident response.

### Helicopter deployment at major incidents

#### Helicopter utilisation during major incidents may include

- Primary and secondary patient transfer
- Transfer of emergency service staff to and between major incident sites
- Deployment and resupply of medical equipment
- Aerial assessment of Major Incident scenes
- Access to remote sites
- Search and rescue

### Helicopter control and safety

- Where multiple emergency services deploy helicopters into an uncontrolled airspace there must be predetermined procedures to coordinate activity and ensure aviation safety
- Organisations operating HEMS must have Standard Operating Procedures (SOPs) for incidents involving multiple HEMS units in uncontrolled airspace that ensure clear command, control, communication and safety
- The safety of HEMS crews should be prioritised when landing in unsurveyed landing sites
- Consider the risk from the cause and consequence of the Major Incident, such as ongoing fire, explosion, toxic chemicals, flooding, weather, building collapse, terrorist activity and secondary attacks directed at emergency responders [13]
- Authority for helicopter deployment should be coordinated by systems familiar with the logistical and clinical capability of the HEMS asset to ensure safe and efficient utilisation
- Contingency plans for regional and supra-regional major incident control centres should be established to mitigate the risk of local command centre communication overload or failure

### HEMS/PHCC teams at major incidents

#### Command and control

- HEMS team may be required to assume medical command of major incident management due to geography, speed of response, medical seniority or predefined Major Incident role
- HEMS teams should contribute to the situational awareness of scene command with intelligence collected from the air and recognise that visual information from flight to and from the scene can be valuable for critical decision making in the scene command
- Deliver regular site report updates to the regional system control centre to allow effective utilisation of available assets
- When possible, HEMS personnel should operate in their usual teams and aviation platforms to minimise risk due to inter service variations in operational procedures
- HEMS teams often have extensive regional knowledge of the health care system and can utilise this knowledge in scene command decision making
- Facilitate scene coordination by wearing labels that clearly state designated Major Incident role and responsibility e.g. Casualty Clearing Station officer, Medical Major Incident Commander etc.
- In accordance with local Major Incident training protocols, responsibility for scene command, safety, communication and safety may take precedence over the personal delivery of medical care to individual casualties

### Triage

- HEMS teams must be able to organise and implement dynamic triage at each stage of the Major Incident response according to national or local triage systems
- HEMS teams must be able to systematically prioritise casualties for treatment and transport. This is a vital element in the efficient management of Major Incidents
- HEMS teams must follow the same triage system as local resources if a national triage system is not in place or when operating in cross border incidents

### Treatment

- Rapid application of simple interventions, such as tourniquet control of massive haemorrhage and recovery position for airway obstruction, may be superior to definitive clinical management of individual patients when managing large numbers of patients with limited resources or in a non permissive environment [8]
- The appropriate level of patient care may change during phases of a Major Incident and as a patient is moved away from direct danger into an increasingly permissive and safe environment
- Implementation of advanced medical procedures, such as intubation, requires situational judgement to ensure that it does not further endanger the individual patient and impair the care delivered to other unattended victims

### Transport

- Decision making on helicopter transfer of patients will be influenced by many event specific factors and may be inappropriate if the helicopter is better deployed to convey staff and equipment. This may change during different phases of the response
- Helicopter evacuation may allow primary transfer to distant specialist care (e.g. burns, extracorporeal membrane oxygenation (ECMO)) or inter-hospital transfers to appropriately distribute patients for definitive care
- HEMS teams may be required to use a knowledge of regional resources and specialist centres to distribute patients according to need and capacity

## EHAC MWG statement

A systematic review of the utilisation of HEMS during the early response to major incidents demonstrated that, in addition to their conventional patient treatment and transport functions, these resources have been used in a diverse range of roles at previous incidents [3]. This diversity reflects the heterogeneous nature of both international HEMS systems and of Major Incidents. During the 7th July 2005 London bombings, despite over 700 patients being triaged or treated by HEMS teams, no patients were transported by helicopter and the aviation resource was used solely as a delivery vector for medical staff and equipment to multiple sites [4]. In other situations helicopters have been used very effectively for primary and secondary transfer of patients to specialist care such as Major Trauma care or ECMO [5, 14].

Effective triage and major incident management by skilled pre hospital physicians has been demonstrated to reduce critical mortality and over triage rates [15]. Appropriate training facilitates rapid and accurate major incident triage [16]. In ballistic or CBRNE situations, triage decisions and the level of medical care delivered will evolve depending on the ongoing threat and it has been proposed that the sophistication of such decisions requires specially trained and equipped senior personnel [17].

## Recovery

### Post incident

- HEMS teams should plan to rapidly regain operational activity to meet conventional need and to respond to further related incidents
- HEMS teams that have been involved in major incident scene management should be relieved when the mission is over to allow for rest, debriefing and psychological support
- Team debriefing and psychological support should be available to HEMS members

### Reporting

- Contemporaneous records of Major Incident events and decision making must be preserved to inform later investigation and analysis and in accordance with local regulations or laws
- Teams should be encouraged to write statements of Major Incident events soon after the incident to promote accurate recording and recollection of events
- A standardised Major Incident reporting template should be completed (http://majorincidentreporting.net)

## EHAC MWG statement

A recent systematic literature review found little or no systematic reporting of the utilisation of HEMS in the early medical management of major incidents [3]. This compromises the opportunity to learn from such events and develop systems that could improve the response to future Major Incidents. Several Major Incident reporting templates have been published [18] and one online template has been developed to specifically assess the pre hospital response [2, 19].

Despite the predominant focus upon minimizing the physical risk to emergency responders at Major Incidents, there is a significant evidence base demonstrating psychopathological sequelae [13]. Critical incident stress debriefing may mitigate the impact of an event [20].
